# The BLI-3/TSP-15/DOXA-1 Dual Oxidase Complex Is Required for Iodide Toxicity in *Caenorhabditis elegans*

**DOI:** 10.1534/g3.114.015982

**Published:** 2014-12-04

**Authors:** Zhaofa Xu, Jintao Luo, Yu Li, Long Ma

**Affiliations:** * State Key Laboratory of Medical Genetics and School of Life Sciences, Central South University, Changsha, Hunan 410078, China; † Institute of Immunology, Shanghai Jiao Tong University School of Medicine, Shanghai 200025, China

**Keywords:** iodide, dual oxidase, reactive oxygen species, H_2_O_2_, *C. elegans*

## Abstract

Iodine is an essential trace element for life. Iodide deficiency can lead to defective biosynthesis of thyroid hormones and is a major cause of hypothyroidism and mental retardation. Excess iodide intake, however, has been linked to different thyroidal diseases. How excess iodide causes harmful effects is not well understood. Here, we found that the nematode *Caenorhabditis elegans* exhibits developmental arrest and other pleiotropic defects when exposed to excess iodide. To identify the responsible genes, we performed a forward genetic screen and isolated 12 mutants that can survive in excess iodide. These mutants define at least four genes, two of which we identified as *bli-3* and *tsp-15*. *bli-3* encodes the *C. elegans* ortholog of the mammalian dual oxidase DUOX1 and *tsp-15* encodes the tetraspanin protein TSP-15, which was previously shown to interact with BLI-3. The *C. elegans* dual oxidase maturation factor DOXA-1 is also required for the arresting effect of excess iodide. Finally, we detected a dramatically increased biogenesis of reactive oxygen species in animals treated with excess iodide, and this effect can be partially suppressed by *bli-3* and *tsp-15* mutations. We propose that the BLI-3/TSP-15/DOXA-1 dual oxidase complex is required for the toxic pleiotropic effects of excess iodide.

Iodine is an essential trace element for life and a key component for the biogenesis of thyroid hormones. Iodine is unevenly distributed in nature. Insufficient intake of iodide is a major cause of thyroid hormone deficiency and can lead to severe developmental defects, including hypothyroidism and mental retardation (Nussey and Whitehead 2001). Mutations in enzymes involved in the generation of H_2_O_2_, the iodination of the thyroglobulin protein, and the recycling of iodide from mono-iodotyrosine and di-iodotyrosine are commonly found in congenital hypothyroidisms (de Vijlder 2003; Moreno *et al.* 2008; Park and Chatterjee 2005; Weber *et al.* 2013).

Although iodide deficiency is a major cause of human diseases, excess iodide intake has severe health consequences as well. Decades ago, Wolff and Chaikoff (1948) found that rats injected with an excess amount of iodide salt could develop an acute symptom that resembles hypothyroidism (the Wolff-Chaikoff effect). Excess iodide intake has been identified as a major risk factor for autoimmune thyroiditis (Bagchi *et al.* 1985; Rose *et al.* 1999; Rose *et al.* 1997; Teng *et al.* 2006), hyperthyroidism (Nussey and Whitehead 2001), hypothyroidism (Rose *et al.* 1999; Teng *et al.* 2006), and thyroid cancers (Blomberg *et al.* 2012; Dong *et al.* 2013; Guan *et al.* 2009; Lind *et al.* 1998). To date, the molecular mechanism that mediates the deleterious effects of excess iodide in humans is largely unknown.

The nematode *Caenorhabditis elegans* has provided a powerful system for dissecting the genetic mechanisms controlling animals’ intrinsic biology and interaction with environmental factors. By studying the *C. elegans* response to excess iodide, we might gain new insights into the biological effects of excess iodide on other animals. In this study, we examined how excess iodide affects *C. elegans* development and identified three genes required for the effects.

## Materials and Methods

### Strains

*C. elegans* strains were grown at 20° as described, unless otherwise indicated (Brenner 1974). N2 (Bristol) was the reference wild-type strain. Strains used in this study include:

CB767 *bli-3(e767) I* (Brenner 1974)
CB769 *bli-1(e769) I* (Brenner 1974)
CB937 *bli-4(e937) I* (Brenner 1974)
SP2275 *tsp-15(sv15) I* (Moribe *et al.* 2004)
CSM421 *duox-2(ok1775) I* (this study, backcrossed five times)
CSM296 *bli-3(mac37) I* (this study)
CSM297 *bli-3(mac38) I* (this study)
CSM299 *bli-3(mac40) I* (this study)
CSM300 *bli-3(mac41) I* (this study)
CSM292 *tsp-15(mac33) I* (this study)
CSM291 *mac32 I* (this study)
CSM294 *mac35 I* (this study)
CSM295 *mac36 I* (this study)
CSM301 *mac42 I* (this study)
CSM302 *mac43 I* (this study)
CSM303 *mac44 I* (this study)
CB768 *bli-2(e768) II* (Brenner 1974)
CB518 *bli-5(e518) III* (Brenner 1974)
MT1655 *bli-6(n776) IV* (Park and Horvitz 1986)
BE16 *bli-6(sc16) IV* (Park and Horvitz 1986)
CSM418 *ZK822.5(ok2281) IV* (this study, backcrossed six times)
CSM298 *mac39 IV* (this study)
CB4856 (Hawaiian) (Wicks *et al.* 2001)

### *C. elegans* survival assay

Five young adults were grown in an OP50-seeded NGM plate supplemented with chemicals (NaI, KI, or NaCl) at different concentrations and their F_1_ progeny were observed. When the concentration of I^−^ is high (*e.g.*, at 5 mM), the F_1_ progeny of wild-type P_0_ animals would arrest at the larval stages and the bacterial food would be barely consumed on day 8. Animals that could survive and propagate in 5 mM I^−^ would generate numerous progeny of mixed developmental stages and consume most or all of the bacterial food by day 8. We found that adding NaI stock solution to a premade NGM agar plate to a final concentration of 5 mM has the same effect on animal development as does a plate to which an equal amount of NaI was added during the preparation of the NGM media.

### Genetic screen and mapping of mutations

Synchronized L4 wild-type animals (P_0_) were mutagenized with EMS (ethyl methanesulfonate) as described (Brenner 1974). F_1_ progeny were allowed to grow to young adults in NGM plates seeded with OP50 bacteria and were transferred to plates containing 5 mM NaI. After 8 d, F_2_ progeny were observed under a dissecting microscope to identify surviving adult animals. From ∼5000 F_1_ (∼10,000 haploid genomes) animals screened, we obtained 12 independent F_2_ isolates.

We mapped the mutations to chromosomes using single nucleotide polymorphisms (SNPs) (Wicks *et al.* 2001) and previously described SNPs (Davis *et al.* 2005). Mutations mapped near each other were tested by genetic complementation for the survival of *trans* heterozygous animals in 5 mM NaI.

### Observation of gonad development, cuticle shedding defect, and intestinal autofluorescence

Bright field pictures of *C. elegans* gonads and cuticle blisters were taken using a digital camera attached to a Leica microscope. Pictures of unshedded cuticles were taken using a digital camera attached to an Olympus SZX16 dissecting microscope. Intestinal autofluorescence of excess iodide-treated animals were observed under an Olympus SZX16 dissecting microscope using either a GFP filter (excitation wavelength 460–495 nm) or an RFP filter (excitation wavelength 530–550 nm).

### Hoechst 33258 staining

Hoechst 33258 staining was performed as described (Moribe *et al.* 2004) with minor modifications. Synchronized young adult animals were washed off plates and incubated at room temperature with gentle shaking for 15 min with 1 μg/ml Hoechst 33258 (Sigma) diluted in M9. After staining, animals were washed three times with M9 and observed under a Leica DM5000B fluorescence microscope.

### Measuring general reactive oxygen species

Whole-animal reactive oxygen species (ROS) production was measured using 2′, 7′-dichlorodihydrofluorescein diacetate (DCFDA) (D6883, Sigma), a fluorescence-base probe for general ROS (Halliwell and Gutteridge 2007) based on a method described previously (Gruber *et al.* 2011) with modifications. Synchronized animals at the L1 larval stage were allowed to recover on NGM agar plates for 8 hr with food and washed three times with H_2_O afterward. Approximately 150 to 300 animals were transferred to each well of a dark-walled 96-well plate containing ample bacterial food, 200 μl H_2_O, and 50 μM DCFDA with or without 5 mM NaI. DCFDA fluorescence intensity was measured using a fluoremeter (Synergy 2; BioTek) at the excitation wavelength of 485 nm and the emission wavelength of 528 nm every 10 min for 12 hr at room temperature. After fluorescence measurement, the exact number of animals in each well was counted. Fluorescence readings were normalized to bacterial controls on the same plate.

We normalized all raw control and experimental values against the mean of the control values (wild-type animals without iodide treatment) of the biological replicates. Statistics were performed using raw values.

### RNA interference

L4 animals were fed HT115 (DE3) bacteria expressing dsRNAs targeting different genes on NGM plates with 1 mM IPTG and 0.1 mg/ml Ampicillin (Timmons *et al.* 2001) with or without 5 mM NaI for 8 d. The progeny were examined under dissecting microscope for survival. The RNAi feeding bacterial strains for *bli-2*, *bli-3*, *bli-5*, *mlt-7*, *skpo-1*, *skpo-2*, *skpo-3*, and *ZK822.5* were obtained from a whole-genome RNAi library (Kamath *et al.* 2003), and the sequences of the plasmid inserts were determined. We generated RNAi constructs targeting *tsp-15*, *doxa-1*, and *F52H2.4* by subcloning a 977-bp *tsp-15* genomic fragment, a 1025-bp *doxa-1* genomic fragment, and an 837-bp *F52H2.4* genomic fragment into the pPD129.36 vector, respectively.

## Results

### Excess iodide causes pleiotropic defects in *C. elegans*

The effect of iodide on the model organism *Caenorhabditis elegans* is largely unknown, except that it can act as an attractant (Ward 1973). To investigate whether iodide affects *C. elegans* development, we examined F_1_ progeny of wild-type young adults grown on OP50-seeded NGM plates containing different concentrations of NaI. We found that at concentrations below 1 mM, NaI had no apparent effect on animal development (Table 1). A stepwise increase of NaI concentrations from 1 mM to 10 mM incrementally suppressed the development of animals. When exposed to high concentrations of NaI, animals never reached adulthood and instead were arrested at or before the L3 (5 mM NaI) or L2 (10 mM NaI) larval stages based on body sizes. This result suggests that NaI has a dose-dependent development-arresting effect on *C. elegans*.

**Table 1:** Dose-dependent effect of iodide on animal development

	Chemicals		
Concentration (mM)	NaI	KI	NaCl
0.1	Starved	Starved	Starved
0.5	Starved	Starved	Starved
1.0	Starved	Starved	Starved
2.0	Scrawny	ND	ND
3.0	L3/YA	ND	ND
4.0	L1/YA	ND	ND
5.0	L1/L3	L1/L3	Starved
10.0	L1/L2	L1/L2	Starved

YA, young adult; ND, not determined.

**Figure 1:**
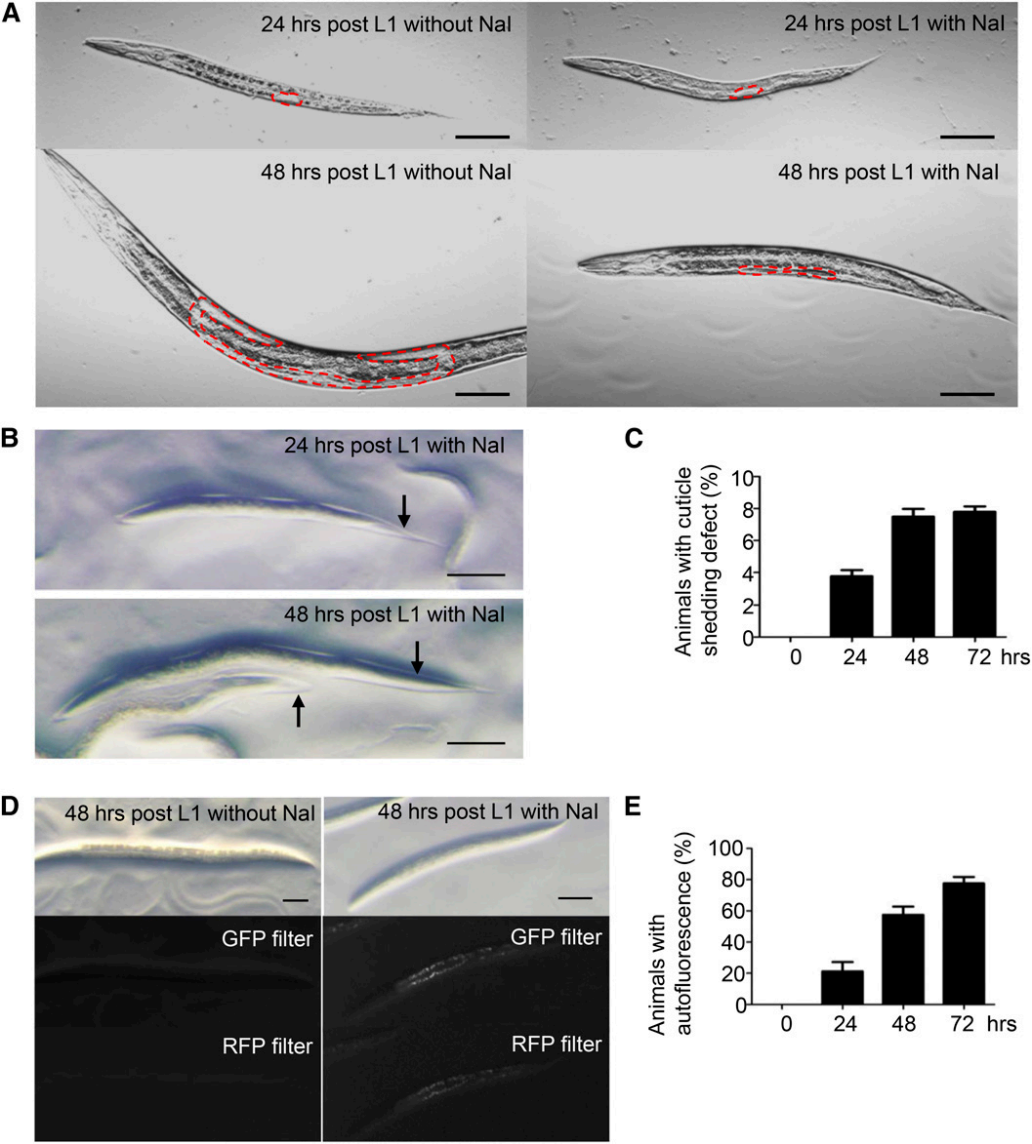
Wild-type animals treated with 5 mM NaI exhibit pleiotropic defects. (A) Gonad development of wild-type animals treated with or without excess iodide. Dotted red lines delineate the gonads. (B) Representative pictures of unshedded old cuticles (arrows) in wild-type animals treated with excess iodide. (C) Quantification of animals with cuticle shedding defects after exposure to excess iodide. (D) Intestinal autofluorescence of animals at 48 hr after exposure to excess iodide. The autofluorescence is obvious under either a GFP or an RFP filter. Left panels show animals without iodide treatment; right panels show animals with iodide treatment. (E) Quantification of animals with intestinal autofluorescence after exposure to excess iodide. Scale bars: 50 μm. For (C) and (E), error bars: SEs of seven biological replicates (n = 90–110 per replicate).

We treated synchronized animals of the L1 larval stage with 5 mM NaI (excess iodide hereafter) and identified the gonads of arrested animals (Figure 1A). We found that the arms of most gonads extended less than halfway along the ventral sides, similar to the morphology of wild-type gonads between the L2 and L3 larval stages (Hirsh *et al.* 1976) and consistent the notion that these animals arrested at or before the L3 larval stage based on body size analysis.

To determine whether Na^+^ plays a role in causing the developmental arrest, we replaced NaI with NaCl in the culture media. At comparable concentrations as that of NaI, NaCl had no apparent effect on animal development, suggesting that Na^+^ does not cause the developmental arrest (Table 1). To understand whether this effect is limited to NaI, we tested KI. At comparable concentrations, KI is equally efficient as NaI in causing the developmental arrest (Table 1).

To examine whether biological activities of the OP50 bacteria might be involved in the development-arresting effect of NaI on *C. elegans*, we fed animals OP50 bacteria killed by heating (75° for 1 hr) and found that excess iodide caused a developmental arrest indistinguishable from that of animals grown on live OP50 (Z. Xu and L. Ma, unpublished observations), suggesting that iodide acts directly on animals. The effect of excess iodide on animal development is reversible, as some arrested larva could resume development and grow to healthy adults when placed in a regular culture plate without NaI (Z. Xu and L. Ma, unpublished observations). Taken together these results suggest that excess iodide is solely responsible for the development-arresting effect on *C. elegans*.

In animals treated with excess iodide, we identified two other apparent defects: a cuticle shedding defect and a premature accumulation of intestinal autofluorescence (Figure 1). The cuticle shedding defect left some animals trapped in old cuticles (Figure 1B, arrows), and approximately 8% of animals had this defect after 48-hr exposure to excess iodide (Figure 1C). Animals exposed to excess iodide also developed strong intestinal autofluorescence (Figure 1D), which reached ∼80% penetrance after 72-hr exposure (Figure 1E). Animals not exposed to NaI rarely exhibited unshedded old cuticles (Z. Xu and L. Ma, unpublished observations) or intestinal autofluorescence (Figure 1D) at comparable time intervals. Therefore, excess iodide caused pleiotropic defects in *C. elegans* that include developmental arrest, cuticle shedding defect, and premature accumulation of intestinal autofluorescence.

### A forward genetic screen identifies mutants that survive in excess iodide

To investigate whether any genes are involved in the development-arresting effect of excess iodide, we mutagenized P_0_ animals with ethyl methanesulfonate and screened the progeny of ∼5000 F_1_ animals for mutants that could survive in 5 mM NaI. We obtained 12 independent isolates from the screen. We mapped all 12 mutations using SNPs (see *Materials and Methods*). Genetic complementation tests suggest that these mutations might affect at least four genes (Table 2).

**Table 2:** List of mutants that can survive in 5 mM NaI

Complementation Group	Isolates	Chromosome
1	*mac****33***	**I**
2	*mac37*, *38*, ***40***, *41*	**I**
3	*mac****32***, *35*, *36*, *42*, *43*, *44*	**I**
4	*mac****39***	**IV**

Alleles in bold are reference alleles.

### Mutants that survive in excess iodide have defective cuticles

**Figure 2:**
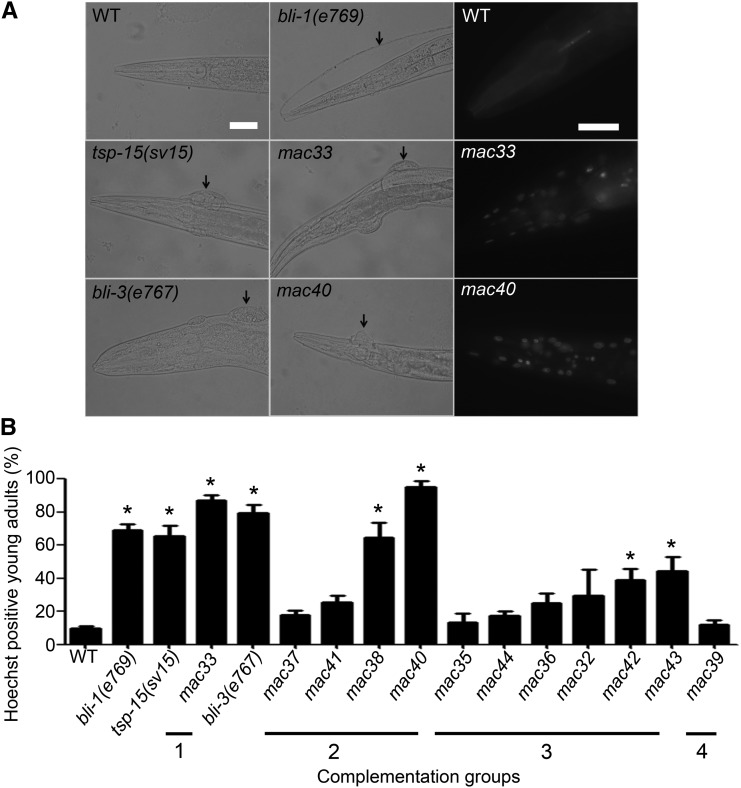
Mutants that survive in 5 mM NaI have defective cuticle integrity. (A) Cuticle blisters (arrows) of *bli* mutants, *tsp-15(sv15)* mutants, and two *mac* mutants (left and middle panels). Hoechst 33258 labels numerous nuclei in *mac33* and *mac40* mutants (right panels). Scale bars: 20 μm. (B) Ratios of Hoechst 33258-positive animals. Statistics: different from wild-type. Bars: SEs of four biological replicates (n = 50 for each replicate). *P < 0.01 (Bonferroni test with one-way ANOVA).

The *mac33* mutation representing complementation group 1 (Table 2) caused an obviously blistered (Bli) phenotype (Figure 2A). The *mac40* mutants of complementation group 2 occasionally exhibited the Bli phenotype as well (Figure 2A). The majority of the mutants, however, did not exhibit an obviously Bli phenotype. To determine whether these mutants had microscopic cuticle lesions that were not obvious under dissecting microscope, we stained animals with the nuclear-binding fluorescence dye Hoechst 33258, which was previously used as a sensitive detection for cuticle integrity (Moribe *et al.* 2004; Thein *et al.* 2009). As shown in Figure 2B, five isolates from complementation groups 1, 2, and 3, including *mac33*, *mac38*, *mac40*, *mac42*, and *mac43*, exhibited defective cuticle integrity (Figure 2A and B), suggesting that these mutants carry genetic lesions affecting *C. elegans* cuticle formation. This result raises the possibility that genes known to affect cuticle biogenesis might be involved in the development-arresting effect of excess iodide.

Mutations affecting *C. elegans* cuticles generally cause several phenotypes that include blisters on cuticle (Bli), dumpy (Dpy), long (Lon), roller (Rol), and squat (Sqt) (Kramer 1997). Because we only detected an obviously Bli phenotype in the *mac* isolates, we postulate that some previously characterized Bli mutants might behave similarly as our isolates for survival in excess iodide. We tested a series of Bli mutants (Table 3), including *bli-1(e769)*, *bli-2(e768)*, *bli-3(e767)*, *bli-4(e937)*, *bli-5(e518)* (Brenner 1974), *bli-6(n776*, *sc16)*, (Park and Horvitz 1986) and *tsp-15(sv15)* (Moribe *et al.* 2004), and found that only *bli-3(e767)* and *tsp-15(sv15)* mutants could survive in excess iodide (Table 3). Hence, a subset of genes involved in cuticle formation is also required for the development-arresting effect of excess iodide.

**Table 3:** Differential effects of excess iodide on the survival of mutants and animals treated with RNAis

Strain	Survival in 5 mM NaI
N2	No
*control RNAi*	No
*bli-1(e769)*	No
*bli-2(e768)*	No
*bli-2(RNAi)*	No
***bli-3(e767)***	**Yes**
***bli-3(RNAi)***	**Yes**
*bli-4(e937)*	No
*bli-5(e518)*	No
*bli-5(RNAi)*	No
*bli-6(sc16)*	No
*bli-6(n776)*	No
***tsp-15(sv15)***	**Yes**
***tsp-15(RNAi)***	**Yes**
***doxa-1(RNAi)***	**Yes**
*duox-2(ok1775)*	No
*mlt-7(RNAi)*	No
*ZK822.5(ok2281)*	No
*ZK822.5(RNAi)*	No
*F52H2.4(RNAi)*	No
*F52H2.4(RNAi) + ZK822.5(RNAi)*	No
*ZK822.5(ok2281)* + *F52H2.4(RNAi)*	No

### Mutations in complementation groups 1 and 2 affect *tsp-15* and *bli-3*, respectively

*bli-3* encodes the *C. elegans* ortholog of the mammalian dual oxidase DUOX1 (Edens *et al.* 2001; Simmer *et al.* 2003), a member of the NOX/DUOX family of NADPH oxidases required for the biogenesis of the reactive oxygen species H_2_O_2_ (Donko *et al.* 2005). BLI-3 oxidase activity is essential for *C. elegans* cuticle formation and pathogen resistance (Chavez *et al.* 2009; Hoeven *et al.* 2011; Moribe *et al.* 2012; Moribe and Mekada 2013; Moribe *et al.* 2004; Zou *et al.* 2013). *tsp-15* encodes a tetraspanin protein homolog that forms a transmembrane protein complex with BLI-3 and the dual oxidase maturation factor DOXA-1 (Moribe *et al.* 2012; Moribe *et al.* 2004). TSP-15 is required for the oxidase activity of BLI-3 (Moribe *et al.* 2012).

Three of our complementation groups were mapped to chromosome I (Table 2), on which both *bli-3* and *tsp-15* locate (www.wormbase.org). Genetic complementation tests between *bli-3(e767)* or *tsp-15(sv15)* and a mutation representing each of the three groups (Table 2, alleles in bold) indicate that *mac40* is an allele of *bli-3* and *mac33* is an allele of *tsp-15*, respectively, whereas *mac32* represents an unknown gene. Consistent with this finding, the blisters of *mac33* and *mac40* mutants are filled with cellular components (Figure 2A) and resemble those of *tsp-15(sv15)* and *bli-3(e767)* mutants but are different from the clear blisters of *bli-1(e769)* mutants (Figure 2A).

**Figure 3:**
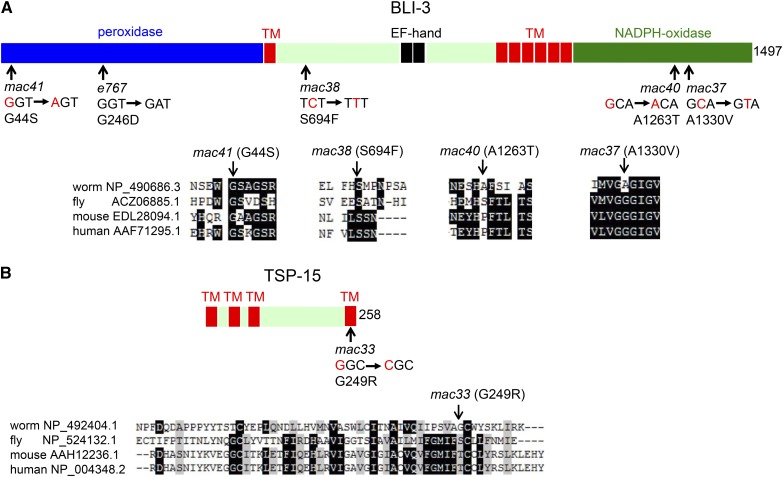
Some *mac* mutations affect *bli-3* and *tsp-15*. (A) The *mac* mutations in *bli-3* affect conserved (*mac41*: G44S, *mac38*: S694F) or nonconserved (*mac40*: A1263T, *mac37*: A1330V) amino acids in different domains of BLI-3. Nucleotide changes, amino acid changes, and BLI-3 DUOX1 partial sequence alignments of different species were presented. TM: transmembrane domain. (B) *mac33* affects a nonconserved amino acid residue in the fourth transmembrane domain of TSP-15. Nucleotide change, amino acid change, and TSP-15 partial sequence alignment were presented.

We determined the coding sequences of *bli-3* in the isolates of the *mac40* complementation group and identified a missense mutation in each of the four mutants (Figure 3A). These mutations caused a G44S (*mac41*) change in the peroxidase domain, an S694F (*mac38*) change in the region between the first transmembrane domain and the EF hand domain, and an A1263T (*mac40*) change and an A1330V (*mac37*) change in the NADPH oxidase domain (Figure 3A).

Similarly in *mac33* mutants, we identified a missense mutation in *tsp-15* that changes a nonconserved Gly249 to Arg in the fourth transmembrane domain of TSP-15 (Figure 3B).

To verify the functions of *bli-3* and *tsp-15* in mediating the arresting effect of excess iodide, we reduced the expression of *bli-3* or *tsp-15* in animals using feeding RNAi. We found that these RNAi-treated animals exhibited the Bli phenotype (Z. Xu and L. Ma, unpublished observations) and acquired the ability for surviving in 5 mM NaI (Table 3). Together with genetic and molecular analyses above, this result suggests that functional losses in *bli-3* or *tsp-15* can suppress the development-arresting effect of excess iodide and the *mac* mutations in *bli-3* or *tsp-15* are loss-of-function mutations.

In addition to developmental arrest, excess iodide also caused cuticle shedding defect and premature accumulation of intestinal autofluorescence in animals (Figure 1). We found that *bli-3(e767)* and *tsp-15(sv15)* mutants treated with excess iodide did not exhibit these phenotypes (Z. Xu and L. Ma, unpublished observations), suggesting that these defects also require the activities of BLI-3 and TSP-15.

### The *C. elegans* dual oxidase maturation factor DOXA-1 is required for the development-arresting effect of excess iodide

DOXA-1 is the *C. elegans* ortholog of the mammalian dual oxidase maturation factor (Grasberger and Refetoff 2006; Morand *et al.* 2009) and forms a complex with BLI-3 and TSP-15 to regulate the biogenesis of H_2_O_2_ (Moribe *et al.* 2012). We found that animals fed dsRNAs targeting *doxa-1* were Bli (Z. Xu and L. Ma, unpublished observations) and could survive in 5 mM NaI (Table 3). Hence, each component of the BLI-3/TSP-15/DOXA-1 dual oxidase complex is required for the development-arresting effect of excess iodide in *C. elegans*.

*C. elegans* cuticle formation also requires MLT-7, an ShkT (Shk toxin)-domain-containing heme peroxidase that crosslinks collagens by reducing H_2_O_2_ generated by the BLI-3 dual oxidase (Thein *et al.* 2009). Animals fed dsRNAs targeting *mlt-7* were Bli (Z. Xu and L. Ma, unpublished observations) but failed to survive in 5 mM NaI (Table 3).

Besides MLT-7, the *C. elegans* genome also encodes three other ShkT-domain-containing peroxidases named SKPO-1, SKPO-2, and SKPO-3 (Tiller and Garsin 2014), among which SKPO-3 is the most identical to MLT-7 (BLAST, www.wormbase.org). SKPO-1 is expressed in the hypodermis and protects *C. elegans* from the infection by bacterial pathogen *Enterococcus faecalis*, whereas SKPO-2 and SKPO-3 do not appear to have such a function (Tiller and Garsin 2014). We examined whether MLT-7 might function redundantly with the SKPO proteins in affecting the survival of animals in excess iodide (Supporting Information, Table S1). We used RNAi to reduce the expression of *mlt-7* and each *skpo* gene individually or in combination. We did not observe survival of these RNAi-treated animals in excess iodide (Table S1). Therefore, MLT-7 and each of the SKPO proteins might not be individually or redundantly required for the development-arresting effect of excess iodide.

In *C. elegans*, *duox-2* encodes a barely expressed paralog of BLI-3 that has a truncated C-terminal NADPH oxidase domain (Edens *et al.* 2001). A *duox-2(ok1775)* deletion mutant failed to survive in excess iodide (Table 3), suggesting that *duox-2* is not required for the development-arresting effect of excess iodide.

Finally, we tested two *C. elegans* genes (*F52H2.4* and *ZK822.5*) that encode homologs (BLAST search, www.wormbase.org) of the mammalian Na/I symporter, a protein that actively transports iodide from the blood into the thyroid gland (Dai *et al.* 1996). We found that a *ZK822.5(ok2281)* deletion mutant (Table 3) and animals treated with *F52H2.4(RNAi)*, *ZK822.5(RNAi)*, or both RNAis (Table 3) were similarly arrested in 5 mM NaI as wild-type animals did, suggesting that *F52H2.4* or *ZK822.5* might not be required for the development-arresting effect of excess iodide.

### Excess iodide promotes ROS biogenesis

Because a conserved function of the BLI-3/TSP-15/DOXA-1 complex is to generate the reactive oxygen species H_2_O_2_ (Moribe *et al.* 2012), we tested whether excess iodide would cause an altered ROS biogenesis, which might contribute to the aforementioned pleiotropic defects. We treated synchronized animals at the L1 larval stage with excess iodide and measured relative ROS biogenesis using 2′, 7′-dichlorodihydrofluorescein diacetate (DCFDA), a fluorescence-base probe for general ROS (Gruber *et al.* 2011; Halliwell and Gutteridge 2007) (see *Materials and Methods*).

**Figure 4:**
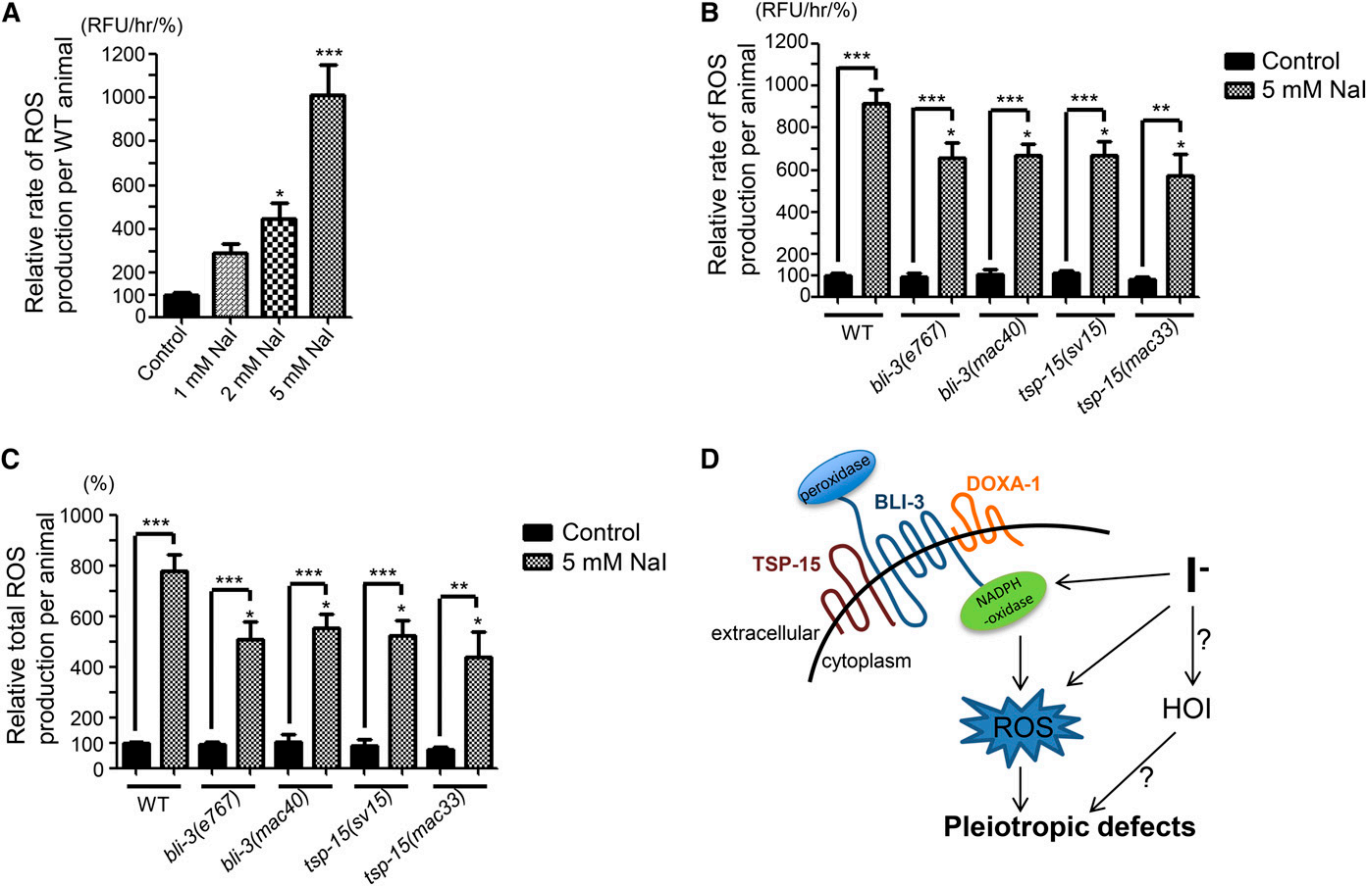
General ROS biogenesis in animals treated with or without 5 mM NaI. (A) Hourly rate of ROS production (relative DCFDA fluorescence intensity unit, RFU) in wild-type animals treated with different concentrations of NaI. Statistics: different from animals without iodide treatment (control). Bars: SEs of nine biological replicates. *P < 0.05, ***P < 0.001 (Bonferroni test with one-way ANOVA). (B) Hourly rate of ROS production in different strains. Without iodide treatment, no significant difference was observed between the wild-type and each *bli-3* or *tsp-15* mutant. With 5 mM NaI treatment, the increase of the rate of ROS production in each mutant strain was significantly less than that in wild-type animals. Statistics: different from wild-type with the same treatment or difference of the same strain with no NaI (control) or 5 mM NaI treatment. Bars: SEs of five biological replicates. *P < 0.05, **P < 0.01, ***P < 0.001 (two-tailed unpaired Student’s *t* test). (C) Total ROS production per animal 12 hr after iodide treatment. Statistics: different from wild-type with the same treatment or difference of the same strain with no NaI (control) or 5 mM NaI treatment. Bars: SEs of five biological replicates. *P < 0.05, **P < 0.01, ***P < 0.001 (two-tailed unpaired Student’s *t* test). (D) A graphic model describing the toxic effect of excess iodide in *C. elegans* (see *Discussion*).

As shown in Figure 4A, with a stepwise increase of the NaI concentration we detected a dose-dependent increase of the rate of ROS production in wild-type animals. It is interesting to note that 2.0 mM NaI caused a more than four-fold increase of the rate of ROS production (Figure 4A). Animals were scrawny but can still survive in 2.0 mM NaI (Table 1), suggesting that *C. elegans* might have a high tolerance for ROS levels.

We next compared the rate of ROS production in the wild-type with that in *bli-3* or *tsp-15* mutant strains. Without iodide treatment, the rate of ROS production in the wild-type was indistinguishable from that in each mutant strain (Figure 4B). This result is consistent with a previous finding that the loss of *bli-3* function did not obviously affect ROS production in *C. elegans* (Chavez *et al.* 2009). With excess iodide, we detected a multiple-fold increase of the rate of ROS production in all strains (Figure 4B). However, the increase in each *bli-3* or *tsp-15* mutant strain (six-fold to seven-fold) was significantly less than that in the wild-type (approximately nine-fold) (Figure 4B). The total production of ROS (normalized DCFDA fluorescence intensity of the last measurement point) (Figure 4C) also showed a significant reduction in each *bli-3* or *tsp-15* mutant strain compared with that in the wild-type. Therefore, *bli-3* and *tsp-15* mutations might partially suppress the increased ROS production caused by excess iodide.

## Discussion

In this study, we suggest that *C. elegans* might be an efficient genetic system for studying the biological effects of excess iodide. We found that excess iodide caused pleiotropic defects in *C. elegans* that include reversible developmental arrest, defective cuticle shedding, premature accumulation of intestinal autofluorescence, and dramatically increased production of ROS. We identified the conserved BLI-3/TSP-15/DOXA-1 dual oxidase complex to be required for the toxic effects of excess iodide.

### BLI-3-dependent ROS production might contribute to iodide-induced ROS biogenesis in *C. elegans*

The increase of iodide-induced ROS production was significantly less in *bli-3* or *tsp-15* mutants than that in wild-type animals (Figure 4, B and C). Nevertheless, excess iodide still caused several-fold increase of ROS production in these mutants, suggesting that BLI-3-independent mechanism(s) might also be involved in iodide-induced ROS biogenesis.

We propose a model to explain the biological effects of excess iodide (Figure 4D). In this model, iodide triggers an increased ROS biogenesis that is partially dependent on activity of the BLI-3/TSP-15/DOXA-1 complex and partially on unknown molecules. Together with other possible iodide forms (*e.g.*, HOI, see below), the increased ROS biogenesis leads to the pleiotropic defects in *C. elegans*. Question marks in the model indicate that we have yet to provide experimental evidence supporting the involvement of HOI in the toxic effects of excess iodide.

### Excess iodide might cause increased ROS biogenesis across species

That excess iodide causes increased ROS biogenesis is not a *C. elegans*-specific phenomenon. For example, several studies showed that treating thyroid tissues or thyrocytes with excess iodide resulted in increased ROS biogenesis (Corvilain *et al.* 2000; Golstein and Dumont 1996; Many *et al.* 1992; Serrano-Nascimento *et al.* 2014; Sharma *et al.* 2008; Vitale *et al.* 2000; Yao *et al.* 2012). Therefore, a coupled increase of ROS biogenesis in response to excess iodide might be a conserved biological phenomenon across species.

The chemical property of iodide (as an electron donor) suggests that it can function as a mild reducing agent. For example, iodide is a scavenger of a variety of ROS in Brown algae of the Laminariales (kelps) and appears to have similar functions in human blood cells (Kupper *et al.* 2008). It is intriguing that ROS biogenesis is dramatically increased in *C. elegans* treated with excess iodide. It is possible that the extra reducing activity of excess iodide might perturb *C. elegans* endogenous redox state, the homeostasis of which is likely essential for animal survival. In response, *C. elegans* increases ROS biogenesis accordingly to rebalance the endogenous redox state. Apparently this hypothesis raises a series of questions, *e.g.*, how a cell detects iodide, how the signals are transmitted, what cells and genes are involved, etc. Future studies are warranted to address these questions.

### Increased ROS biogenesis and other factors might function together to cause iodide-induced pleiotropic defects in *C. elegans*

Our findings suggest that the dramatically increased ROS biogenesis might be a major cause of the pleiotropic defects in animals treated with excess iodide, as functional losses in the BLI-3/TSP-15/DOXA-1 complex could significantly reduce iodide-induced ROS biogenesis and suppress these defects. However, we could not exclude the possibility that other iodide forms, molecules, or pathways are also involved in mediating the toxic effects of excess iodide.

It was shown that iodide could be catalyzed by peroxidases into the toxic hypoiodous acid (HOI) *in vitro* (Magnusson *et al.* 1984a, b). HOI has bactericidal and antiviral activity (Fischer *et al.* 2011; Klebanoff 1967) and could inactivate proteins by iodination (Philpot and Small 1939). The mammalian airway mucosa uses dual oxidase-generated H_2_O_2_ to convert iodide to HOI by lactoperoxidase-catalyzed oxidation (Fischer *et al.* 2011). The *C. elegans* genome encodes two close homologs of lactoperoxidase (Gotenstein *et al.* 2010) and several other similar proteins (Tiller and Garsin 2014), raising the possibility that in *C. elegans* iodide might be converted to HOI as well. Therefore, the toxicity of excess iodide could be a combined consequence of excess HOI and ROS *in vivo* (see Figure 4D).

*C. elegans* cuticle formation requires activities of the H_2_O_2_-generating BLI-3/TSP-15/DOXA-1 dual oxidase complex, the MLT-7 peroxidase that reduces H_2_O_2_ to crosslink cuticle collagens, and the participation of numerous extracellular matrix components (Edens *et al.* 2001; Moribe *et al.* 2012; Page and Johnstone 2007; Thein *et al.* 2009). Functional losses in the BLI-3 dual oxidase complex or the MLT-7 peroxidase could cause cuticle crosslinking defects (Edens *et al.* 2001; Moribe *et al.* 2012; Thein *et al.* 2009). However, little is known about the effects of functional gains in these proteins. Excess iodide can cause a dramatically increased ROS biogenesis in *C. elegans*, which might mimic a functional gain in BLI-3 activity and shift the collagen-crosslinking reaction catalyzed by the MLT-7 peroxidase toward over-crosslinking. Over-crosslinked cuticles might not be degraded during molting, resulting in unshedded cuticles in animals treated with excess iodide.

Is it possible that *bli-3*, *tsp-15*, or *doxa-1* mutants survive in excess iodide because the mutants have defective cuticles and therefore can shed them more easily? We found that *bli-1*, *bli-2*, *bli-4*, *bli-5*, *bli-6*, and *mlt-7* mutants and/or RNAi-treated animals all had defective cuticles and were still arrested by excess iodide (Table 3). Therefore, the cuticle defect is not sufficient for these animals to survive in excess iodide.

### The *in vivo* function of iodine in *C. elegans* remains to be understood

Wild *C. elegans* animals are found in gardens, compost piles, and rotting fruits (Felix and Braendle 2010; Kiontke and Sudhaus 2006), and might have access to iodine by feeding on iodine-containing bacteria or environmental iodide compounds. The *C. elegans* genome contains genes encoding sodium/iodide symporter-like proteins (see above) and an ortholog of the iodotyrosine deiodinase (IYD) (de la Cruz *et al.* 2014; Friedman *et al.* 2006; Gnidehou *et al.* 2004; Moreno 2003), suggesting that iodide might function biologically in the animal. Iodide is not an essential ingredient in the standard *C. elegans* culture medium (Brenner 1974). However, the agar, a phycocolloid extracted from marine algae (Hitchens and Leikind 1939) used for growing the bacterial food (Brenner 1974) and other medium ingredients might contain residual iodide that is sufficient for animals to survive. Future studies might answer the question whether iodine is essential for *C. elegans* development.

In short, we provide evidence that *C. elegans* could serve as an efficient genetic system for studying the biological effect of excess iodide. We found that excess iodide has toxic pleiotropic effects on *C. elegans* and could cause a dramatic increase of *in vivo* ROS biogenesis. The BLI-3/TSP-15/DOXA-1 dual oxidase complex is required for the toxicity and is partially responsible for the increased ROS biogenesis. We suggest that genes interacting with the BLI-3/TSP-15/DOXA-1 dual oxidase complex might be identified, *e.g.*, by screening for mutants that can survive in or are hypersensitive to excess iodide.

## Supplementary Material

Supporting Information

## References

[bib1] Bagchi N. Brown T. R. Urdanivia E. Sundick R. S., 1985 Induction of autoimmune thyroiditis in chickens by dietary iodine. Science 230: 325–327. 4048936 10.1126/science.4048936

[bib2] Blomberg M. Feldt-Rasmussen U. Andersen K. K. Kjaer S. K., 2012 Thyroid cancer in Denmark 1943–2008, before and after iodine supplementation. Int. J. Cancer 131: 2360–2366. 22337133 10.1002/ijc.27497

[bib3] Brenner S., 1974 The genetics of *Caenorhabditis elegans*. Genetics 77: 71–94. 4366476 10.1093/genetics/77.1.71 PMC1213120

[bib4] Chavez V. Mohri-Shiomi A. Garsin D. A., 2009 Ce-Duox1/BLI-3 generates reactive oxygen species as a protective innate immune mechanism in *Caenorhabditis elegans*. Infect. Immun. 77: 4983–4989. 19687201 10.1128/IAI.00627-09 PMC2772517

[bib5] Corvilain B. Collyn L. van Sande J. Dumont J. E., 2000 Stimulation by iodide of H(2)O(2) generation in thyroid slices from several species. Am. J. Physiol. Endocrinol. Metab. 278: E692–E699. 10751204 10.1152/ajpendo.2000.278.4.E692

[bib6] Dai G. Levy O. Carrasco N., 1996 Cloning and characterization of the thyroid iodide transporter. Nature 379: 458–460. 8559252 10.1038/379458a0

[bib7] Davis M. W. Hammarlund M. Harrach T. Hullett P. Olsen S., 2005 Rapid single nucleotide polymorphism mapping in *C. elegans*. BMC Genomics 6: 118. 16156901 10.1186/1471-2164-6-118 PMC1242227

[bib8] de la Cruz I. P. Ma L. Horvitz H. R., 2014 The *Caenorhabditis elegans* iodotyrosine deiodinase ortholog SUP-18 functions through a conserved channel SC-Box to regulate the muscle two-pore domain potassium channel SUP-9. PLoS Genet. 10: e1004175. 24586202 10.1371/journal.pgen.1004175 PMC3930498

[bib9] de Vijlder J. J., 2003 Primary congenital hypothyroidism: defects in iodine pathways. Eur. J. Endocrinol. 149: 247–256. 14514339 10.1530/eje.0.1490247

[bib10] Dong W. Zhang H. Zhang P. Li X. He L., 2013 The changing incidence of thyroid carcinoma in Shenyang, China before and after universal salt iodization. Med. Sci. Monit. 19: 49–53. 23314590 10.12659/MSM.883736 PMC3629016

[bib11] Donko A. Peterfi Z. Sum A. Leto T. Geiszt M., 2005 Dual oxidases. Philos. Trans. R. Soc. Lond. B Biol. Sci. 360: 2301–2308. 16321800 10.1098/rstb.2005.1767 PMC1569583

[bib12] Edens W. A. Sharling L. Cheng G. Shapira R. Kinkade J. M., 2001 Tyrosine cross-linking of extracellular matrix is catalyzed by Duox, a multidomain oxidase/peroxidase with homology to the phagocyte oxidase subunit gp91phox. J. Cell Biol. 154: 879–891. 11514595 10.1083/jcb.200103132 PMC2196470

[bib13] Felix M. A. Braendle C., 2010 The natural history of *Caenorhabditis elegans*. Curr. Biol. 20: R965–R969. 21093785 10.1016/j.cub.2010.09.050

[bib14] Fischer A. J. Lennemann N. J. Krishnamurthy S. Pocza P. Durairaj L., 2011 Enhancement of respiratory mucosal antiviral defenses by the oxidation of iodide. Am. J. Respir. Cell Mol. Biol. 45: 874–881. 21441383 10.1165/rcmb.2010-0329OC PMC3208616

[bib15] Friedman J. E. Watson J. A. Jr Lam D. W. Rokita S. E., 2006 Iodotyrosine deiodinase is the first mammalian member of the NADH oxidase/flavin reductase superfamily. J. Biol. Chem. 281: 2812–2819. 16316988 10.1074/jbc.M510365200

[bib16] Gnidehou S. Caillou B. Talbot M. Ohayon R. Kaniewski J., 2004 Iodotyrosine dehalogenase 1 (DEHAL1) is a transmembrane protein involved in the recycling of iodide close to the thyroglobulin iodination site. FASEB J. 18: 1574–1576. 15289438 10.1096/fj.04-2023fje

[bib17] Golstein J. Dumont J. E., 1996 Cytotoxic effects of iodide on thyroid cells: difference between rat thyroid FRTL-5 cell and primary dog thyrocyte responsiveness. J. Endocrinol. Invest. 19: 119–126. 8778164 10.1007/BF03349847

[bib18] Gotenstein J. R. Swale R. E. Fukuda T. Wu Z. Giurumescu C. A., 2010 The *C. elegans* peroxidasin PXN-2 is essential for embryonic morphogenesis and inhibits adult axon regeneration. Development 137: 3603–3613. 20876652 10.1242/dev.049189 PMC2964093

[bib19] Grasberger H. Refetoff S., 2006 Identification of the maturation factor for dual oxidase. Evolution of an eukaryotic operon equivalent. J. Biol. Chem. 281: 18269–18272. 16651268 10.1074/jbc.C600095200

[bib20] Gruber J. Ng L. F. Fong S. Wong Y. T. Koh S. A., 2011 Mitochondrial changes in ageing *Caenorhabditis elegans*–what do we learn from superoxide dismutase knockouts? PLoS ONE 6: e19444. 21611128 10.1371/journal.pone.0019444 PMC3097207

[bib21] Guan H. Ji M. Bao R. Yu H. Wang Y., 2009 Association of high iodine intake with the T1799A BRAF mutation in papillary thyroid cancer. J. Clin. Endocrinol. Metab. 94: 1612–1617. 19190105 10.1210/jc.2008-2390

[bib22] Halliwell B. Gutteridge J. M. C., 2007 Free radicals in biology and medicine, Oxford University Press, Oxford, New York.

[bib23] Hirsh D. Oppenheim D. Klass M., 1976 Development of the reproductive system of *Caenorhabditis elegans*. Dev. Biol. 49: 200–219. 943344 10.1016/0012-1606(76)90267-0

[bib24] Hitchens A. P. Leikind M. C., 1939 The Introduction of Agar-agar into Bacteriology. J. Bacteriol. 37: 485–493. 16560221 10.1128/jb.37.5.485-493.1939 PMC374482

[bib25] Hoeven R. McCallum K. C. Cruz M. R. Garsin D. A., 2011 Ce-Duox1/BLI-3 generated reactive oxygen species trigger protective SKN-1 activity via p38 MAPK signaling during infection in *C. elegans*. PLoS Pathog. 7: e1002453. 22216003 10.1371/journal.ppat.1002453 PMC3245310

[bib26] Kamath R. S. Fraser A. G. Dong Y. Poulin G. Durbin R., 2003 Systematic functional analysis of the *Caenorhabditis elegans* genome using RNAi. Nature 421: 231–237. 12529635 10.1038/nature01278

[bib27] Kiontke K. Sudhaus W., 2006 Ecology of *Caenorhabditis* species. *WormBook*, ed. The *C. elegans* Research Community, WormBook, /10.1895/wormbook.1.37.1. Available at http://www.wormbook.org. 10.1895/wormbook.1.37.1 PMC4780885 18050464

[bib28] Klebanoff S. J., 1967 Iodination of bacteria: a bactericidal mechanism. J. Exp. Med. 126: 1063–1078. 4964565 10.1084/jem.126.6.1063 PMC2138423

[bib29] Kramer J. M., 1997 Extracellular Matrix, C. elegans II, edited by Riddle D. L. Blumenthal T. Meyer B. J. Priess J. R., Cold Spring Harbor Laboratory Press, Cold Spring Harbor, NY. ISBN-10: 0-87969-532-3.

[bib30] Kupper F. C. Carpenter L. J. McFiggans G. B. Palmer C. J. Waite T. J., 2008 Iodide accumulation provides kelp with an inorganic antioxidant impacting atmospheric chemistry. Proc. Natl. Acad. Sci. USA 105: 6954–6958. 18458346 10.1073/pnas.0709959105 PMC2383960

[bib31] Lind P. Langsteger W. Molnar M. Gallowitsch H. J. Mikosch P., 1998 Epidemiology of thyroid diseases in iodine sufficiency. Thyroid 8: 1179–1183. 9920375 10.1089/thy.1998.8.1179

[bib32] Magnusson R. P. Taurog A. Dorris M. L., 1984 a Mechanism of iodide-dependent catalatic activity of thyroid peroxidase and lactoperoxidase. J. Biol. Chem. 259: 197–205. 6706930

[bib33] Magnusson R. P. Taurog A. Dorris M. L., 1984 b Mechanisms of thyroid peroxidase- and lactoperoxidase-catalyzed reactions involving iodide. J. Biol. Chem. 259: 13783–13790. 6094529

[bib34] Many M. C. Mestdagh C. van den Hove M. F. Denef J. F., 1992 *In vitro* study of acute toxic effects of high iodide doses in human thyroid follicles. Endocrinology 131: 621–630. 1639011 10.1210/endo.131.2.1639011

[bib35] Morand S. Ueyama T. Tsujibe S. Saito N. Korzeniowska A., 2009 Duox maturation factors form cell surface complexes with Duox affecting the specificity of reactive oxygen species generation. FASEB J. 23: 1205–1218. 19074510 10.1096/fj.08-120006 PMC2660643

[bib36] Moreno J. C., 2003 Identification of novel genes involved in congenital hypothyroidism using serial analysis of gene expression. Horm. Res. 60(Suppl 3): 96–102. 14671405 10.1159/000074509

[bib37] Moreno J. C. Klootwijk W. van Toor H. Pinto G. D’Alessandro M., 2008 Mutations in the iodotyrosine deiodinase gene and hypothyroidism. N. Engl. J. Med. 358: 1811–1818. 18434651 10.1056/NEJMoa0706819

[bib38] Moribe H. Konakawa R. Koga D. Ushiki T. Nakamura K., 2012 Tetraspanin is required for generation of reactive oxygen species by the dual oxidase system in *Caenorhabditis elegans*. PLoS Genet. 8: e1002957. 23028364 10.1371/journal.pgen.1002957 PMC3447965

[bib39] Moribe H. Mekada E., 2013 Co-occurrence of tetraspanin and ROS generators: Conservation in protein cross-linking and other developmental processes. Worm 2: e23415. 24058871 10.4161/worm.23415 PMC3704445

[bib40] Moribe H. Yochem J. Yamada H. Tabuse Y. Fujimoto T., 2004 Tetraspanin protein (TSP-15) is required for epidermal integrity in *Caenorhabditis elegans*. J. Cell Sci. 117: 5209–5220. 15454573 10.1242/jcs.01403

[bib41] Nussey, S., and S. Whitehead, 2001 in *Endocrinology: An Integrated Approach*, Oxford. 20821847

[bib42] Page A. P. Johnstone I. L., 2007 The cuticle. *WormBook*, ed. The *C. elegans* Research Community, WormBook, /10.1895/wormbook.1.138.1. Available at http://www.wormbook.org.

[bib43] Park E. C. Horvitz H. R., 1986 Mutations with dominant effects on the behavior and morphology of the nematode *Caenorhabditis elegans*. Genetics 113: 821–852. 3744028 10.1093/genetics/113.4.821 PMC1202915

[bib44] Park S. M. Chatterjee V. K., 2005 Genetics of congenital hypothyroidism. J. Med. Genet. 42: 379–389. 15863666 10.1136/jmg.2004.024158 PMC1736062

[bib45] Philpot J. S. Small P. A., 1939 The actions of iodine and hypoiodous acid on pepsin. Biochem. J. 33: 1727–1733. 16747089 10.1042/bj0331727 PMC1264637

[bib46] Rose N. R. Rasooly L. Saboori A. M. Burek C. L., 1999 Linking iodine with autoimmune thyroiditis. Environ. Health Perspect. 107(Suppl 5): 749–752. 10502541 10.1289/ehp.99107s5749 PMC1566262

[bib47] Rose N. R. Saboori A. M. Rasooly L. Burek C. L., 1997 The role of iodine in autoimmune thyroiditis. Crit. Rev. Immunol. 17: 511–517. 9419438

[bib48] Serrano-Nascimento C. da Silva Teixeira S. Nicola J. P. Nachbar R. T. Masini-Repiso A. M., 2014 The acute inhibitory effect of iodide excess on sodium/iodide symporter expression and activity involves the PI3K/Akt signaling pathway. Endocrinology 155: 1145–1156. 24424051 10.1210/en.2013-1665

[bib49] Sharma R. Traore K. Trush M. A. Rose N. R. Burek C. L., 2008 Intracellular adhesion molecule-1 up-regulation on thyrocytes by iodine of non-obese diabetic.H2(h4) mice is reactive oxygen species-dependent. Clin. Exp. Immunol. 152: 13–20. 18241232 10.1111/j.1365-2249.2008.03590.x PMC2384069

[bib50] Simmer F. Moorman C. van der Linden A. M. Kuijk E. van den Berghe P. V., 2003 Genome-wide RNAi of *C. elegans* using the hypersensitive *rrf-3* strain reveals novel gene functions. PLoS Biol. 1: E12. 14551910 10.1371/journal.pbio.0000012 PMC212692

[bib51] Teng W. Shan Z. Teng X. Guan H. Li Y., 2006 Effect of iodine intake on thyroid diseases in China. N. Engl. J. Med. 354: 2783–2793. 16807415 10.1056/NEJMoa054022

[bib52] Thein M. C. Winter A. D. Stepek G. McCormack G. Stapleton G., 2009 Combined extracellular matrix cross-linking activity of the peroxidase MLT-7 and the dual oxidase BLI-3 is critical for post-embryonic viability in *Caenorhabditis elegans*. J. Biol. Chem. 284: 17549–17563. 19406744 10.1074/jbc.M900831200 PMC2719394

[bib53] Tiller G. R. Garsin D. A., 2014 The SKPO-1 Peroxidase Functions in the Hypodermis to Protect *Caenorhabditis elegans* From Bacterial Infection. Genetics 197: 515–526. 24621828 10.1534/genetics.113.160606 PMC4063911

[bib54] Timmons L. Court D. L. Fire A., 2001 Ingestion of bacterially expressed dsRNAs can produce specific and potent genetic interference in *Caenorhabditis elegans*. Gene 263: 103–112. 11223248 10.1016/s0378-1119(00)00579-5

[bib55] Vitale M. Di Matola T. D’Ascoli F. Salzano S. Bogazzi F., 2000 Iodide excess induces apoptosis in thyroid cells through a p53-independent mechanism involving oxidative stress. Endocrinology 141: 598–605. 10650940 10.1210/endo.141.2.7291

[bib56] Ward S., 1973 Chemotaxis by the nematode *Caenorhabditis elegans*: identification of attractants and analysis of the response by use of mutants. Proc. Natl. Acad. Sci. USA 70: 817–821. 4351805 10.1073/pnas.70.3.817 PMC433366

[bib57] Weber G. Rabbiosi S. Zamproni I. Fugazzola L., 2013 Genetic defects of hydrogen peroxide generation in the thyroid gland. J. Endocrinol. Invest. 36: 261–266. 23404134 10.3275/8847

[bib58] Wicks S. R. Yeh R. T. Gish W. R. Waterston R. H. Plasterk R. H., 2001 Rapid gene mapping in *Caenorhabditis* elegans using a high density polymorphism map. Nat. Genet. 28: 160–164. 11381264 10.1038/88878

[bib59] Wolff J. Chaikoff I. L., 1948 Plasma inorganic iodide as a homeostatic regulator of thyroid function. J. Biol. Chem. 174: 555–564. 18865621

[bib60] Yao X. Li M. He J. Zhang G. Wang M., 2012 Effect of early acute high concentrations of iodide exposure on mitochondrial superoxide production in FRTL cells. Free Radic. Biol. Med. 52: 1343–1352. 22330063 10.1016/j.freeradbiomed.2012.02.002

[bib61] Zou C. G. Tu Q. Niu J. Ji X. L. Zhang K. Q., 2013 The DAF-16/FOXO transcription factor functions as a regulator of epidermal innate immunity. PLoS Pathog. 9: e1003660. 24146615 10.1371/journal.ppat.1003660 PMC3798571

