# Supplementary material for: The BLI-3/TSP-15/DOXA-1 Dual Oxidase Complex Is Required for Iodide Toxicity in Caenorhabditis elegans
Source: G3 (Bethesda). 2014 Dec 4;5(2):195–203. doi: 10.1534/g3.114.015982 (PMC4321028; doi:10.1534/g3.114.015982)
Supplement: Supporting Information [file supp_5_2_195__index.html]

The BLI-3/TSP-15/DOXA-1 Dual Oxidase Complex Is Required for Iodide Toxicity in Caenorhabditis elegans — Supporting Information 

# The BLI-3/TSP-15/DOXA-1 Dual Oxidase Complex Is Required for Iodide Toxicity in *Caenorhabditis elegans*

## Supporting Information for Xu *et al.*, 2015

**Files in this Data Supplement:**

- Table S1 - Survival of wild-type animals treated with feeding RNAis targeting *mlt-7* and each *skpo* gene individually or in combination. (PDF, 153 KB)
