# Supplementary material for: The BLI-3/TSP-15/DOXA-1 Dual Oxidase Complex Is Required for Iodide Toxicity in Caenorhabditis elegans
Source: G3 (Bethesda). 2014 Dec 4;5(2):195–203. doi: 10.1534/g3.114.015982 (PMC4321028; doi:10.1534/g3.114.015982)
Supplement: Supporting Information [file supp_g3.114.015982_015982SI.pdf]

G3·Genes | Genomes | Genetics

The BLI-3/TSP-15/DOXA-1 dual oxidase complex is required for iodide toxicity in *Caenorhabditis elegans*

Zhaofa Xu[1], Jintao Luo[1], Yu Li[2], Long Ma[1*]

1. State Key Laboratory of Medical Genetics and School of Life Sciences, Central South University, Changsha, Hunan 410078, China
2. Institute of Immunology, Shanghai Jiao Tong University School of Medicine, Shanghai 200025, China

*Corresponding author (e-mail: malong@sklmg.edu.cn)

DOI: 10.1534/g3.114.015982

Table S1 Survival of wild-type animals treated with feeding RNAis targeting *mlt-7* and each *skpo* gene individually or in combination.

Strain	Survival (5 mM NaI)
control RNAi	No
bli-3(RNAi)	**Yes**
mlt-7(RNAi)	No
skpo-1(RNAi)	No
skpo-2(RNAi)	No
skpo-3(RNAi)	No
skpo-1(RNAi) + skpo-2(RNAi) + skpo-3(RNAi)	No
skpo-1(RNAi) + mlt-7(RNAi)	No
skpo-2(RNAi) + mlt-7(RNAi)	No
skpo-3(RNAi) + mlt-7(RNAi)	No
skpo-1(RNAi) + skpo-2(RNAi) + skpo-3(RNAi) + mlt-7(RNAi)	No
